# Identification of a Polyketide Synthase Required for Alternariol (AOH) and Alternariol-9-Methyl Ether (AME) Formation in *Alternaria alternata*

**DOI:** 10.1371/journal.pone.0040564

**Published:** 2012-07-06

**Authors:** Debjani Saha, Ramona Fetzner, Britta Burkhardt, Joachim Podlech, Manfred Metzler, Ha Dang, Christopher Lawrence, Reinhard Fischer

**Affiliations:** 1 Department of Microbiology, Karlsruhe Institute of Technology (KIT) - South Campus, Institute for Applied Biosciences, Karlsruhe, Germany; 2 Department of Food Chemistry, Karlsruhe Institute of Technology (KIT) - South Campus, Institute for Applied Biosciences, Karlsruhe, Germany; 3 Karlsruhe Institute of Technology (KIT) - South Campus, Institute of Organic Chemistry, Karlsruhe, Germany; 4 Virginia Bioinformatics Institute, Department of Biological Sciences, Virginia Tech, Blacksburg, Virginia, United States of America; University of Wisconsin – Madison, United States of America

## Abstract

*Alternaria alternata* produces more than 60 secondary metabolites, among which alternariol (AOH) and alternariol-9-methyl ether (AME) are important mycotoxins. Whereas the toxicology of these two polyketide-based compounds has been studied, nothing is known about the genetics of their biosynthesis. One of the postulated core enzymes in the biosynthesis of AOH and AME is polyketide synthase (PKS). In a draft genome sequence of *A. alternata* we identified 10 putative PKS-encoding genes. The timing of the expression of two PKS genes, *pksJ* and *pksH*, correlated with the production of AOH and AME. The PksJ and PksH proteins are predicted to be 2222 and 2821 amino acids in length, respectively. They are both iterative type I reducing polyketide synthases. PksJ harbors a peroxisomal targeting sequence at the C-terminus, suggesting that the biosynthesis occurs at least partly in these organelles. In the vicinity of *pksJ* we found a transcriptional regulator, *altR*, involved in *pksJ* induction and a putative methyl transferase, possibly responsible for AME formation. Downregulation of *pksJ* and *altR* caused a large decrease of alternariol formation, suggesting that PksJ is the polyketide synthase required for the postulated Claisen condensations during the biosynthesis. No other enzymes appeared to be required. PksH downregulation affected *pksJ* expression and thus caused an indirect effect on AOH production.

## Introduction

**Figure 1:**
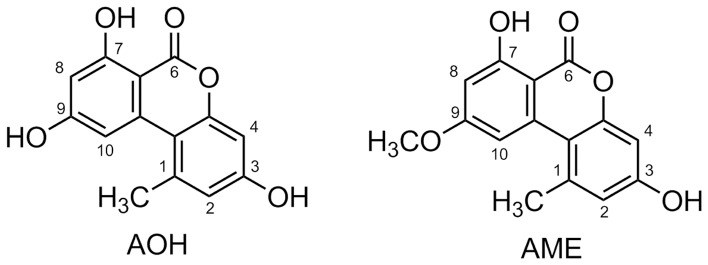
Structure of alternariol (AOH) and alternariol-9-methyl ether (AME).

Alternariol (AOH) and alternariol-9-methyl ether (AME) (**Fig. 1**) are major toxins produced by species within the fungal genus *Alternaria* and are common contaminants of food such as cereals, fruits and fruit juices [1], [2]. AOH exhibits cytotoxic, foetotoxic and teratogenic effects and is suspected to be mutagenic and also associated with the etiology of oesophageal cancer [3]. AOH causes weak acute toxic effects and the LD50 is higher than 400 mg/kg of body weight for mice. AOH is lethal to unborn mice at levels of 100 mg/kg b.w. [4]. It has been reported that AOH induces lipid peroxidation in the epithelium of the fetal esophagus *in vitro* [3]. There are several reports that AOH and AME exhibit genotoxic potentials, e.g. induction of DNA strand breaks, gene mutations in cultured human and animal cells [5], [6], and inhibition of topoisomerase I and IIα under cell free conditions [7]. Recently, it has been shown that AOH and AME are readily hydroxylated by hepatic microsomes from rat, pig and human [6]. The absorption and metabolism of AOH and AME has also been demonstrated *in vitro* with Caco-2 cells, a human epithelial colorectal adenocarcinoma cell line [8].

The predominant classes of fungal secondary metabolites include polyketides, non-ribosomal peptides, terpenes, and alkaloids [9]. Alternariol (AOH) is thought to be formed by the polyketide route of biosynthesis, which is a common pathway for the formation of many fungal secondary metabolites [10], [11]. Fungal polyketide synthases (PKSs) are crucial for the first steps of the biosynthesis of several mycotoxins and other secondary metabolites. Fungal polyketides are produced by multi-domain type I PKSs, which are iterative in nature. Fungal PKSs can be further grouped into non-reducing (NR), partially reducing (PR) and highly reducing (HR) PKSs according to their domain organization [12]. NR-PKS usually contain a starter unit ACP transacylase (SAT), β-ketoacyl synthase (KS), acyl transferase (AT), product template (PT), acyl carrier protein (ACP) and claisen-cyclase/thiolesterase (CLC/TE) domains. In some cases the NR-PKS also harbor methyl transferase (MeT) and reductase (R) domains. PR-PKSs contain KS, AT, dehydratase (DH), ketoreductase (KR) and terminate with ACP. HR-PKSs, on the other hand, contain KS, AT, and DH domains. In many cases DH is followed by MeT, enoyl reductase (ER), KR and a terminating ACP domain. The iterative nature of fungal PKSs means that in the majority of cases only one PKS is involved in the biosynthesis of a particular fungal polyketide.

Biosynthetic routes for AOH were first extensively studied by Thomas [13] who suggested that this metabolite might be synthesized by head-to-tail condensations of acetate units. Later, Gatenbeck and Hermodsson [14] determined that malonate formed by carboxylation of acetate was the polycondensing molecule. These authors also isolated an enzyme, alternariol-O-methytransferase from *A. alternata* that contained O-methyltransferase activity, which converted AOH to AME [15].

Since the discovery of different mycotoxins and their deleterious properties to humans and animals, efforts have been directed toward the understanding of the molecular mechanisms leading to their biosynthesis. To date, the most studied and well characterized mycotoxin biosynthesis pathways are for aflatoxin and sterigmatocystin (sterigmatocystin is the penultimate precursor of aflatoxin) [16]. Genes required for the synthesis of aflatoxin are well conserved among Aspergilli and are located in large gene clusters [17]. The relative order and transcriptional direction of some of the homologous gene pairs though are not conserved [18]. Thus far, most of the genes in the respective clusters have been shown to encode enzymes required for toxin biosynthesis [19], [20]. In addition, the cluster harbors a transcription factor, AflR, which controls the expression of many or all genes located in the aflatoxin gene cluster [21]. In addition, the expression control requires specific chromatin remodeling of the clusters and thus epigenetic control [22], [23]. This appears to be also true for other fungi [24].

Although much progress has been made on the molecular characterization of the genes involved in and the regulation of the biosynthesis of other mycotoxins like fumonisin or trichothecene, no report is yet available regarding alternariol biosynthesis. The genes involved in AOH biosynthesis have yet to be discovered, despite AOH being one of the major mycotoxins produced by Alternaria species such as *A*. *alternata* and despite the importance of AOH as contaminant of food and feed.

Here we present the first report on a polyketide synthase involved in the biosynthesis of alternariol (AOH) and alternariol-methyl-ether (AME). We used both, gene deletion and RNA-silencing strategies to knock-down the function of PKS and other genes in *A. alternata*, which are involved in the biosynthetic pathway.

## Results

### Characterization of polyketide synthase genes (PKS) in *A. alternata*

**Figure 2:**
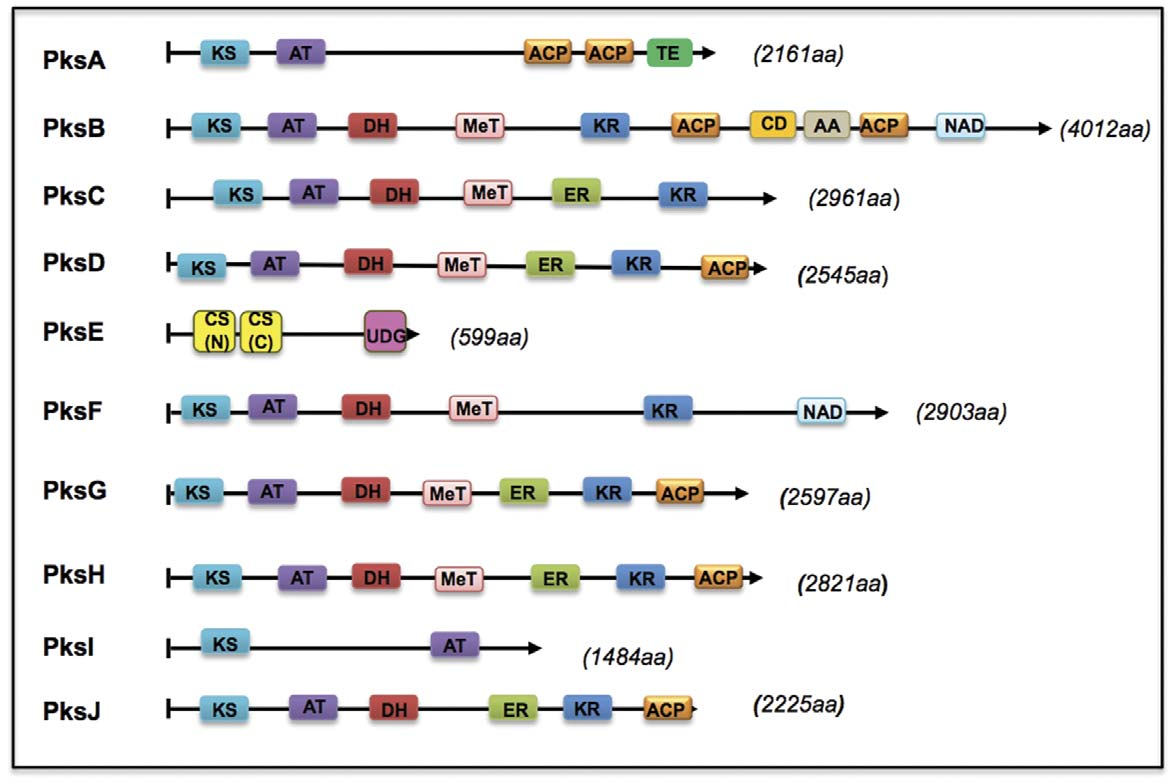
Architecture of PKSs of *Alternaria alternata*. KS, β-ketoacyl synthase; AT, acyltransferase; DH, dehydratase; MT, methyltransferase; ER, enoyl reductase; KR, ketreductase; ACP, acyl carrier protein, CD, condensation domain; AA, Amino acid adenylation domain; CS:Chalon- and Stilben-Synthase (N)/(C); UDG: Uracil DNA Glycolase Superfamily; NAD, NAD binding domain. The sequences of the PKS loci are deposited under the following accession numbers: *pksA* (JX103636); *pksB* (JX103637); *pksC* (JX103638); *pksD* (JX103639); *pksE* (JX103640); *pksF* (JX103641); *pksG* (JX103642); *pksH* (JX103643); *pksI* (JX103644); *pksJ* (JX103645). The genbank accession numbers are given in brackets.

Our aim was to identify the genes encoding enzymes involved in alternariol (AOH) biosynthesis. Because it is likely that the pathway contains a polyketide synthase as one of the central enzymes, we screened an *A. alternata* genome sequence for PKS-encoding genes. The *A. alternata* genome has been sequenced recently (Lawrence et al., unpublished) using 454 Titanium deep sequencing technology (Roche, Indianapolis, IN). An approximate 20× draft of the ∼32 Mb genome sequence was assembled using Newbler software (Roche, Indianapolis, IN) and was used to search for PKS genes in this species. The analysis of the complete genome sequence will be published elsewhere. Applying blast searches with amino acid sequences of 50 known polyketide synthases from different fungi identified 10 putative polyketide synthases in *A. alternata*. Intron-exon borders were predicted using FGENESH (softberry.com) trained on *A. brassicicola* gene models but have not yet been verified experimentally due to the rather large nature of PKS genes. Among these 10 PKS genes, the PKS involved in melanin biosynthesis was already known and named ALM (albino) [25]. We renamed ALM according to a three-letter standard abbreviation code for genes, as PksA, and the remaining ones as PksB to PksJ. As the architectures of fungal PKSs are very similar to each other, we first analyzed the domain structures of the PKSs of *A. alternata* with SMART, WoLFPSORT, epestfind, ELM, ScanProsite and InterPro Scan software tools using standard parameters (**Fig. 2**). Except *pksE*, all PKS genes encode iterative type I RD-PKSs. Typical RD-PKS conserved domains, such as KR, DH and ER, were identified in PksC, PksD, PksF, PksG, PksH and PksJ in addition to KS, AT and ACP except PksC and PksF, which lack the ACP domain. Surprisingly the *pksI* gene is predicted to encode a protein of only 1484 amino acids, containing a KS and an AT domain. PksE did not show any known domain of fungal polyketide synthases. However, the 599 aa long protein of *pksE* displays similarity to chalcone (CHS)/stilbene (STS) synthase and uracil DNA glycosylase like domains. The CHS/STS domain of PksE shares 40% identity to type III polyketide synthase of *Sordaria macrospora* [26]. Type III PKS are relatively small dimeric proteins (subunit sizes about 40–45 kDa) that usually carry out iterative condensation reactions with malonyl-CoA; the numbers can range from one to seven. CHS and STS are plant-specific polyketide synthases. CHS catalyzes the first step in the biosynthesis of a large number of biologically important substances, e.g. flavonoids (flower color) and phytoalexins (defense against pathogens). STS forms the backbone of the stilbene phytoalexins. However, these enzymes are rare in higher plants.

**Figure 3:**
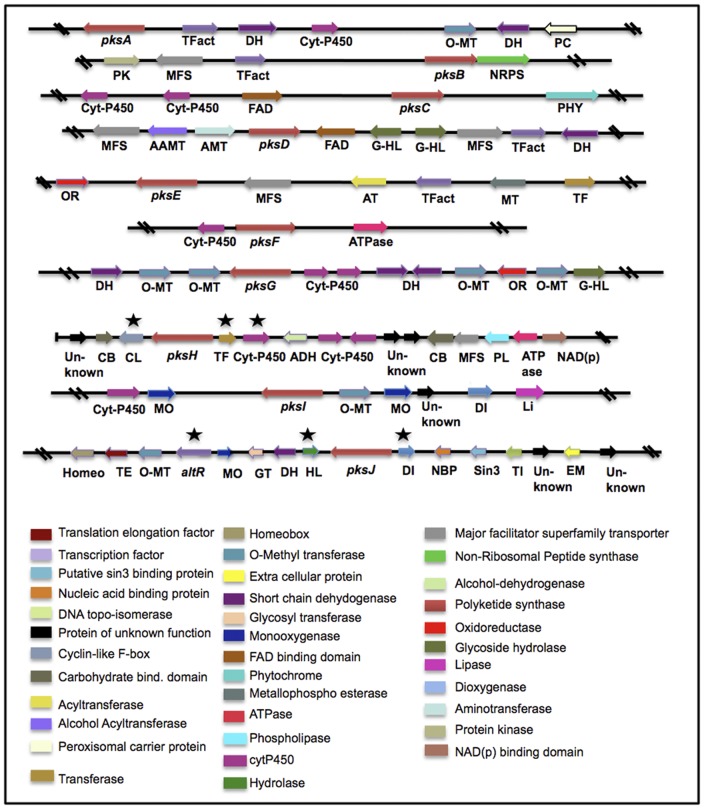
Organization of polyketide biosynthesis gene clusters in *Alternaria alternata*. Each arrow indicates the direction of transcription deduced from the analysis of the nucleotide sequences. The asterisks indicate the genes of the *pksJ* and *pksH* clusters, which have been silenced in addition to *pksJ and pksH*.

As mentioned above, genes encoding enzymes involved in the biosynthesis of a given secondary metabolite are usually clustered in the genome. Together with other biosynthetic enzymes, a regulatory transcription factor is often found within the cluster. In order to better understand the roles and organization of PKS biosynthetic clusters in *A. alternata* we analyzed the genome sequences up- and downstream of the PKSs. Indeed most of the gene clusters also contained putative transcription factors (**Fig. 3**).

**Figure 4:**
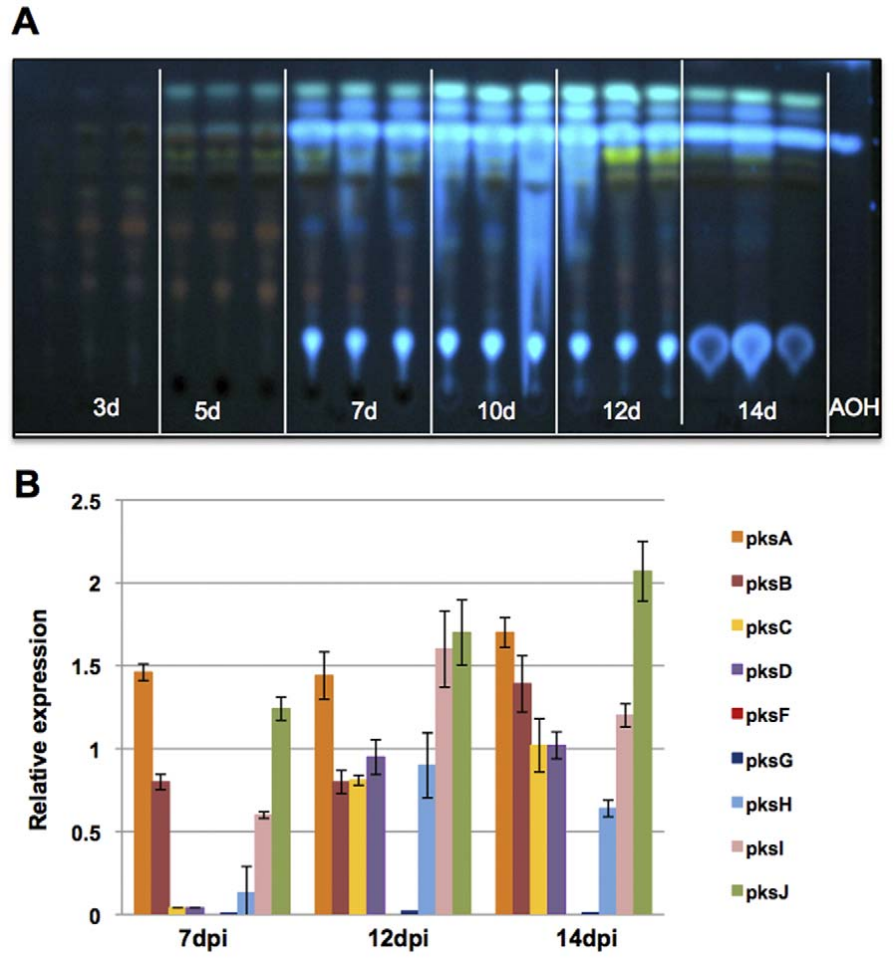
Time-course expression analysis of different PKS genes. (**A**) TLC analysis of AOH formation at different time points. Extracts from cultures grown on MCDB agar at 28°C in constant darkness for 3, 5, 7, 10, 12 and 14 days, respectively. For each time point three extractions were performed. The last lane contained the AOH standard. (**B**) Quantitative real time reverse-transcription polymerase chain reaction (RT-PCR) gene expression analysis of different PKS genes after 7, 12 and 14 days of post inoculation (dpi).

Secondary metabolites are often not produced during logarithmic growth of fungi, but rather in aging mycelium or in response to certain stimuli [27]. The induction of the biosynthesis is probably a result of differential gene expression under those conditions. Therefore, we determined temporal aspects of when individual PKS genes were expressed and correlated this expression pattern with the timing of AOH production. As a first step to identify the initial time point for AOH production, MCDB agar plates were inoculated with defined numbers of spores and incubated for 3 to 14 days at 28°C in the dark. Both, mycelium and agar medium were extracted with ethyl acetate and analyzed by thin layer chromatography (TLC). AOH was detected initially after 5 days in very small quantities and increased after 7 days of incubation (**Fig. 4A**). In order to determine the relative expression levels of the selected PKS genes, RNA was isolated from the same mycelium samples at three different time points, starting from day 7 and subjected to real time PCR analyses (**Fig. 4B**). Except *pksE*, all other PKS genes were expressed by 12 days, making them potential candidates for being involved in AOH biosynthesis.

### Analysis of alternariol biosynthesis through down-regulation

**Figure 5:**
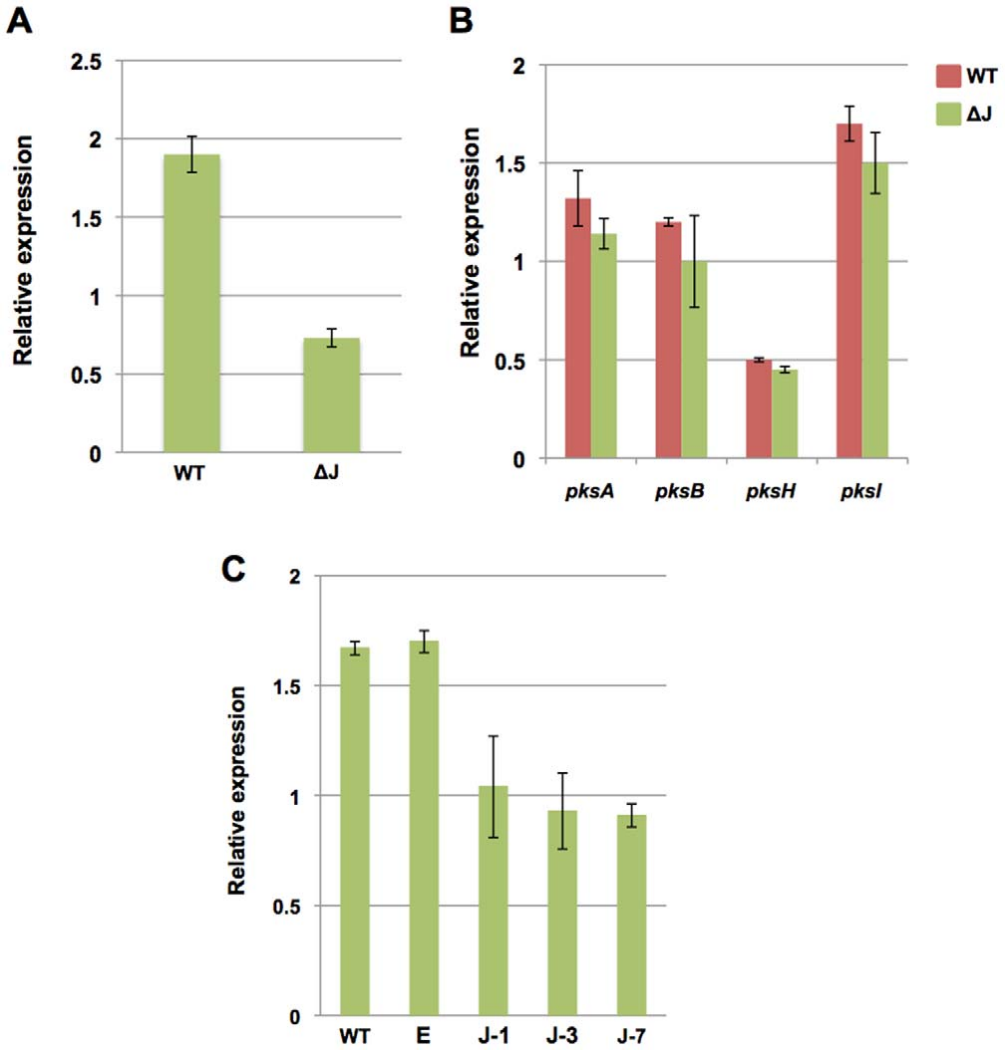
Quantitative PCR analysis of total RNA from the *pksJ* transformant relative to wildtype, using *benA* as reference gene. Both the wildtype and transformants were grown on liquid MCDB medium for 12 days at 28°C in constant darkness. (**A**) *pksJ* transcript level of the wildtype and *pksJ* transformant (ΔJ, heterokaryotic *pksJ* deletion). (**B**) Relative expression of selected PKS transcripts in wildtype and transformant (ΔJ). (**C**) Real time RT-PCR detection of *pksJ* transcripts in wildtype (WT), empty vector control (E) and the silenced transformants J1, J3, J7.

**Figure 6:**
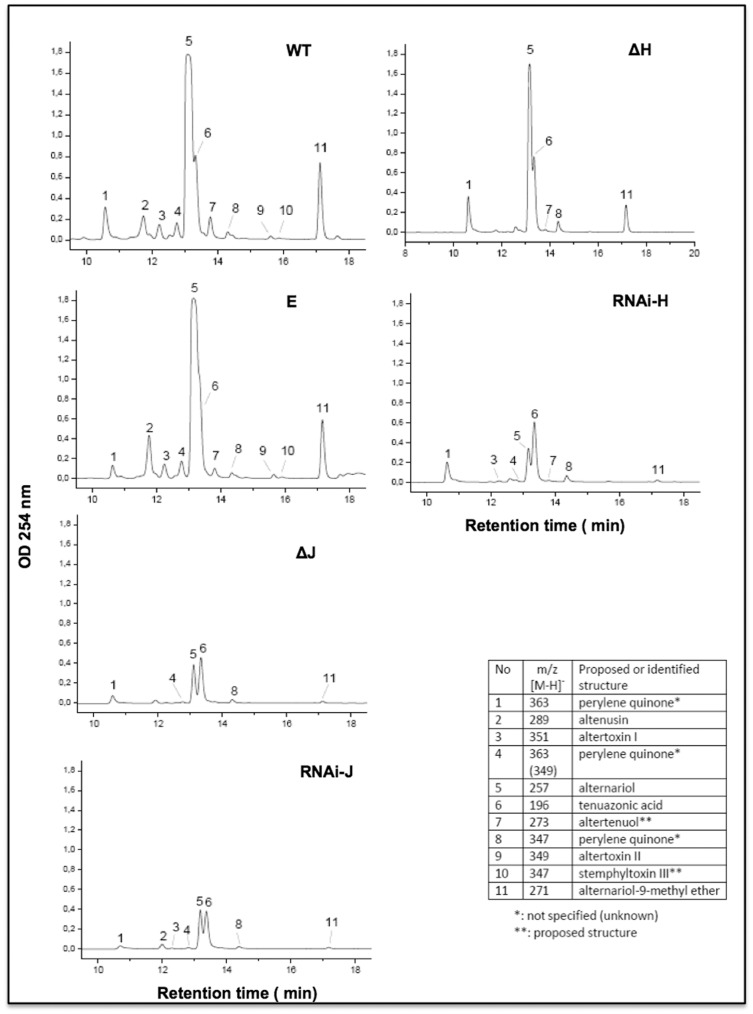
LC/MS analysis of metabolites produced by *Alternaria alternata* WT, WT transformed with an empty vector (E), the Δ*pksJ* strains, the RNAi-*pksJ* strain, the *ΔpksH* and the RNAi-*pksH* strains as detected by UV absorbance. m/z values are given for the most prominent peaks. Peak no 5 corresponds to alternariol and peak no 11 correspond to alternariol-9-methyl ether.

To initiate the molecular analysis of alternariol (AOH) production in *A. alternata* we aimed to identify the role of *pksJ* by gene deletion, as the *pksJ* gene showed the highest expression among all PKSs from day 7 onwards. Approximately, one kb upstream (left border) and downstream regions (right border) of the predicted *pksJ* ORF were PCR amplified and fused to the hygromycin B (*hph*) resistance cassette by fusion PCR [28]. The 5.7 kb long fusion PCR product was directly used for protoplast transformation and homologous replacement of the *pksJ* open reading frame. Transformants were analyzed by PCR and Southern blotting (data not shown). The integration of the construct was verified by PCR using primers derived from the hygromycin B cassette and primers outside the left or the right border sequences. Southern blot analysis with a probe for hygromycin B confirmed the results and demonstrated a single integration (data not shown). However, a wildtype copy of *pksJ* was still detectable by PCR and Southern blotting in the transformants. Even though we performed multiple rounds of single spore isolation on hygromycin-containing media, we anticipated that the transformants still harbored non-transformed wildtype nuclei as a heterokaryon. The other possibility of multiple copies in the genome was excluded because there was only one band detected in the Southern blot using wildtype genomic DNA. Bioinformatic analysis of the genome also revealed only one copy of this gene. In addition to single spore isolation to purify the mutants, several rounds of protoplast isolation were performed, where protoplasts were generated and re-grown to single colonies, but no effect was observed concerning the existence of the wildtype band. Because gene dosage would most likely result in reduced mRNA levels, we compared the *pksJ* mRNA levels in the transgenic strain with wildtype. Total RNA was harvested from wildtype and the heterokaryotic *pksJ-*deletion strain grown in MCBD liquid culture for 12 days as described above and real time RT-PCR analysis was performed. The *pksJ* mRNA expression of the transformant was indeed down regulated approximately 60% in comparison to wildtype (**Fig. 5A**). Next, the transformants were tested for AOH production by TLC. We inoculated MCDB agar plates in the way described above and incubated them for 12 days. Both, mycelium and agar medium were extracted with ethyl acetate and analyzed visually as well as by LC/MS. AOH production was greatly reduced in the transformants compared to wildtype as visualized on TLC. A ∼50-fold reduction in alternariol levels in the transformant (0.66 nmol/10 µl) compared to wildtype (34 nmol/10 µl) and almost no AME production was determined by LC/MS metabolic profiling (**Fig. 6**). To assure that down-regulation of *pksJ* caused the reduction of AOH and AME formation directly and not through an effect on the expression of other PKS genes, we examined the expression of several *pks* genes in wildtype and the transformant. qRT-PCR analyses confirmed no significant difference in the expression of *pksA*, *pksB*, *pksH* and pks*I* (**Fig. 5B**). These results suggest that *pksJ* encodes a PKS directly responsible for the production of AOH and AME.

Because we could not obtain a monokaryotic *pksJ*-deletion strain, we confirmed the role of *pksJ* by RNA silencing. RNA silencing has already been successfully applied for the functional analysis of the *actT2* gene in *A. alternata* [29]. The RNA silencing vector expressing hairpin *pkaJ* RNA, constructed by sense and antisense sequences of *pksJ* (500 bp) in pSilent-1 carrying *hygB* (*hph*) as the selection marker, was transformed into *A. alternata* protoplasts as described previously. The sequence used to construct the hairpin RNA corresponds to the β-ketoacyl synthase region of *pksJ* and the specificity of the sequence was confirmed by genome sequence analysis. The empty pSilent-1 vector was used as a control. After several rounds of purification of the transformants, integration of the *pksJ* RNA silencing vector was analyzed by PCR amplification of vector specific regions taking the primer set corresponding to the hairpin head and sense part of the dsRNA (data not shown). Transformants were examined for production of AOH by TLC and 3 transformants showing lower AOH production were selected for further analysis (data not shown). Because RNA silencing in plants and animals has been described as being due to posttranscriptional degradation of targeted mRNA, we tested whether the low level of AOH correlated with a lower level of *pksJ* mRNA accumulation in these transformants. Total RNA was isolated from both, the wildtype and the transformants, followed by qRT-PCR. Total RNA isolated from a transformant obtained with the empty pSilent-1 vector was also included in the real time RT-PCR analysis. As shown in **Fig. 5C**, *pksJ* was highly expressed in wildtype and the control strain transformed with the empty vector, but the expression was lowered by ∼45% in the *pksJ* transformant. However, this level of suppression was sufficient to disturb the PKS biosynthesis machinery, since LC/MS analysis of the silenced strains depicted a similar result as obtained with the *pksJ*-deletion strain (**Fig. 6**).

### Identification of a PKS affecting *pksJ* expression

It has been already reported in some cases, that two different fungal PKSs could be involved in the synthesis of a single polyketide; such as a set of two unusual type I multifunctional PKSs for the biosynthesis of lovastatin and compactin in *Aspergillus terreus* and *Penicillium citrinum*, respectively. These fungi carry an unusual PKS gene cluster for the cholesterol lowering polyketides in which two pks genes (*lovB* and *lovF* of *A. terreus*, or *mlcA* and *mlcB* of *P. citrinum*) are closely linked in the cluster and are required for the biosynthesis of nonaketide and diketide moieties, respectively [30]. Another example in which two different PKS genes are required for the biosynthesis of a single polyketide is the T-toxin of *Cochliobolus heterostrophus* [31]. Zearalenone production in *Gibberella zeae* also requires two different polyketide synthase genes (*pks13* and *pks14*) [32]. Very recently, it has been also reported by targeted gene deletion studies, that two separate gene clusters are also required for the biosynthesis of meroterpenoids austinol and dehydroaustinol in *A. nidulans* [33]. One is a cluster of four genes including a polyketide synthase gene, *ausA*. The second is a cluster on a separate chromosome comprised of 10 genes including a prenyltransferase gene.

**Figure 7:**
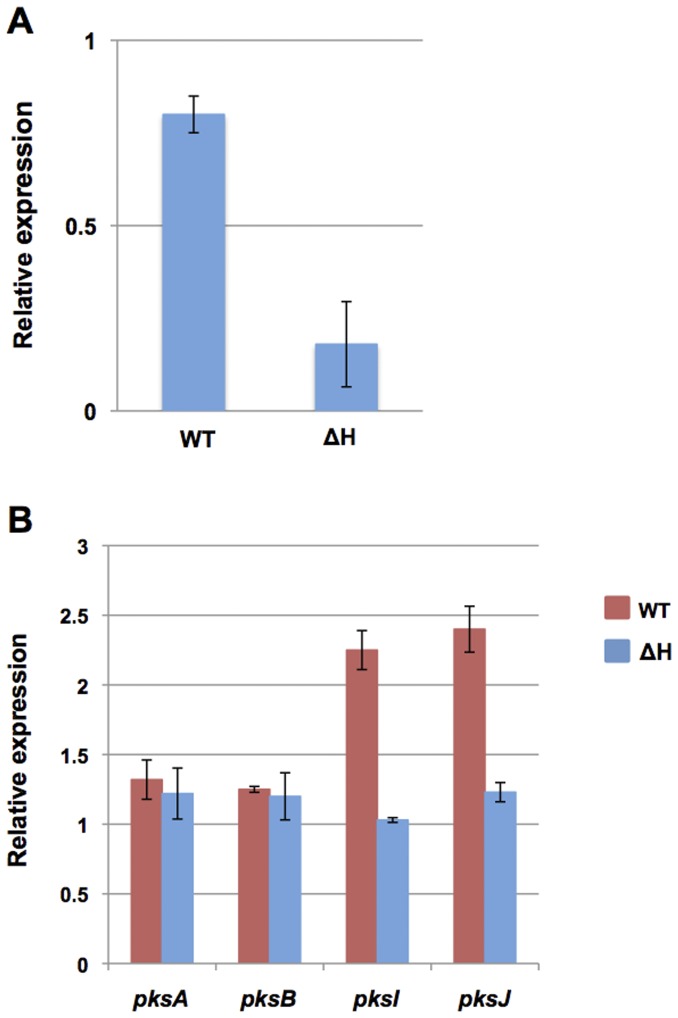
Quantitative PCR analysis of total RNA from the *pksH* transformant (ΔH, heterokaryotic *pksH* deletion) relative to wildtype, using *benA* as reference gene. Both the wildtype and transformants were grown on liquid MCDB medium for 12 days at 28°C in constant darkness. (**A**) *pksH* transcript level of the wildtype and transformant. (**B**) Relative expression of selected *pks* transcripts in wildtype and *pksH* transformant.

In order to test, whether any additional PKSs might be required for alternariol biosynthesis, we applied RNAi silencing to knock down the remaining PKSs. *pksB*, and *pksI* down-regulation did not affect the alternariol level, whereas downregulation of *pksH* affected AOH and AME production. The expression analysis by real time RT-PCR revealed almost 60% downregulation of *pksH* mRNA in the transformants (data not shown)(three transformants were analyzed). The down-regulation lowered AOH and AME production to almost 98%. To verify the result, we also tried to delete *pksH*. We were able to isolate strains with the homologous integration event, but as observed before, the strain still harbored the wildtype copy of the gene even after several rounds of purification. However, real time RT-PCR revealed that the *pksH* mRNA level was reduced to 20% in comparison to wildtype in the transformant (**Fig. 7A**). LC/MS analyses also confirmed the reduction with lower AOH and AME production.

Next, we examined the expression of some PKSs in the *pksH*-downregulated transformant by real time RT-PCR. In comparison to wildtype, the expression of *pksJ* and *pksI* were reduced to almost 50%, while the other PKS genes remained unchanged (**Fig. 7B**). This suggests that *pksH* affects *pksJ* and *pksI* expression and through down-regulation of *pksJ* affects AOH biosynthesis.

### Identification of additional genes for alternariol biosynthesis

**Figure 8:**
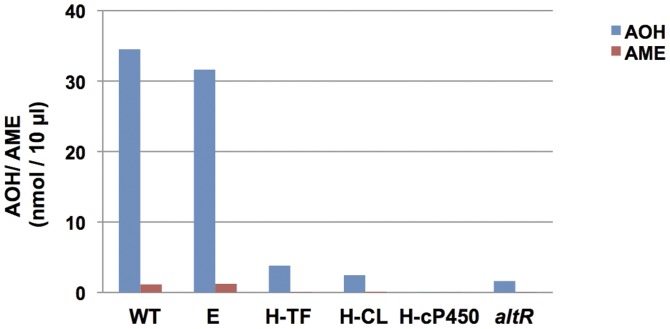
LC/MS analysis of alternariol (AOH) and alternariol-9-methyl ether (AME) formation in wildtype (WT), empty vector (E) and RNAi transformant of selected genes flanked by *pksJ* and *pksH*. H-TF:PksH-transferase; H-CL: PksH-cyclin F-Box; H-cP450: PksH-cytochrome p450; *altR* ( = alternariol regulator). AOH and AME values are expressed in nmol/10 µl.

Next we aimed to identify additional genes involved in AOH biosynthesis. Facilitating the search, secondary metabolism genes in fungi are usually clustered, prompting us to focus on the genes surrounding *pksJ* and *pksH*. We functionally analyzed three genes of the *pksJ* gene cluster (**Fig. 3**), a gene with homology to fungal specific transcription factors (*altR* = alternariol regulator); a gene with homology to phosphoserine phosphatase with haloacid dehalogenase-like hydrolases putative conserved domain (J-HL) and a gene with homology to phytonyl CoA dioxygenase (J-DI). Ten mutants were analyzed for J-DI and J-HL, whereas 6 mutants were selected for *altR*. RNAi silencing of the dioxygenase and the hydrolase gene did not produce a significant difference in AOH and AME production when compared to wildtype by LC/MS analysis (data not shown); indicating that these genes are probably not required for AOH biosynthesis. However, in the RNAi knockdown mutant of the putative transcription factor *altR*, its mRNA level was reduced to almost 50% compared to wildtype (data not shown) and AOH and AME formation was significantly reduced (**Fig. 8**).

Similarly as for the *pksJ* cluster analysis, we analyzed three genes in the *pksH* gene cluster; a gene encoding a protein with homology to cytochrome P450 (H-cP450), a protein with homology to transferase (H-TF) and a protein containing a putative cyclin like F-box domain (H-CL). RNAi mediated silencing of these genes (six mutants were tested for each gene) gave a chemotype similar to that previously characterized with *pksJ* and *pksH* transformants, i.e, little production of AOH and AME (**Fig. 8**). This suggests that all three genes are probably involved in the formation of a secondary metabolite possibly regulating *pksJ* expression.

## Discussion

Polyketides synthesized by fungi display a remarkably rich diversity of structural motifs and accompanying biological activities [9], [34]. Although much progress has been made on the molecular characterization of the genes involved in the biosynthesis of various mycotoxins such as aflatoxin, sterigmatocystin, fumonisin, trichothecene, there are only few reports on alternariol (AOH) biosynthesis and the control of the production [35], [36]. Despite being one of the major mycotoxins formed by *A. alternata* and despite its importance in food and feed contamination, genes involved in AOH biosynthesis were not yet discovered prior to this study. Previous genetic studies in *A. alternata* were mainly associated with host-specific toxins (HSTs) [37], [38]. Pathogenic *A. alternata* strains include different pathotypes, each of which has a distinct and limited host range, characterized by the production of host-specific toxins (HSTs) essential for pathogenesis [39]. The mechanism of host-selective pathogenesis, through the HSTs, is well understood and about 20 HSTs have been documented [40], of which at least seven are from *A. alternata* pathotypes [41].

**Figure 9:**
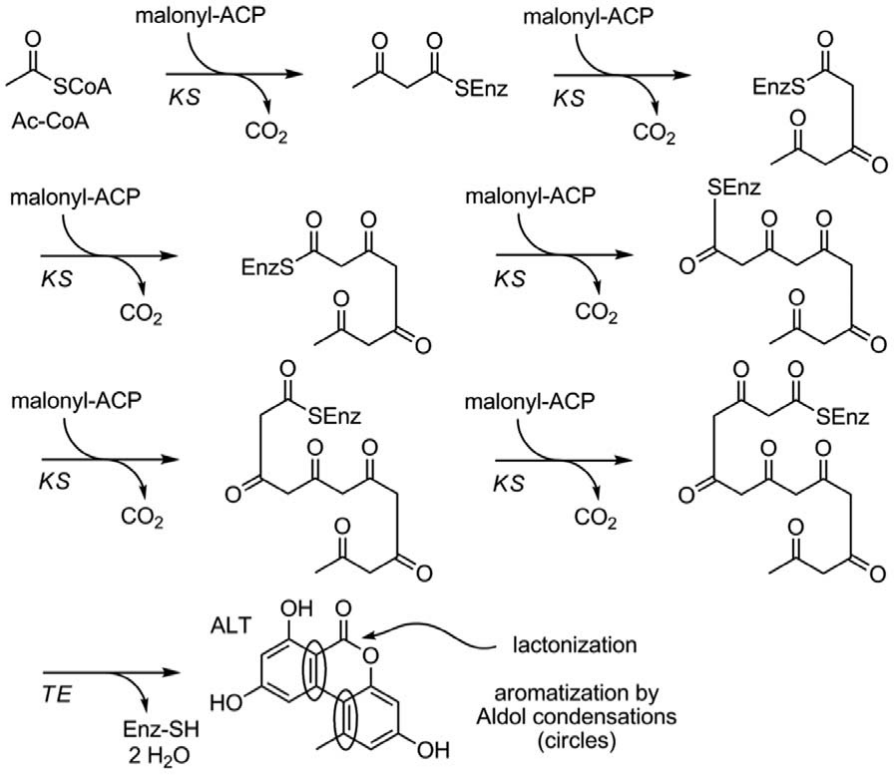
Proposed biosynthetic pathway for alternariol and alternariol-9-methyl ether. The biosynthesis of alternariol and alternariol-9-methyl ether consists of Claisen-type condensations with malonate as building blocks.

The biosynthesis of AOH and thus of its methylated derivative alternariol-9-methyl ether (AME) should be exceptionally simple (**Fig. 9**). It has been noted before that it might consist of Claisen-type condensations with malonate as building blocks without the necessity of any reduction and/or elimination steps [42], [43]. Consequently only the domains catalyzing the Claisen condensations, namely ketosynthases are necessary, of course together with the mandatory acyl carrier proteins (ACP) carrying the malonyl moieties and a thioesterase needed for the finalization of the sequence. The obvious biosynthesis starts with acetyl-CoA and consists of six Claisen condensations, in each of which activated malonate is integrated with concomitant loss of carbonate. Since only two ketosynthase domains participating in alternariol biosynthesis have been identified, it is likely that these condensations are catalyzed by the same domain. Aromatization leading to the final natural product is possible before or after the liberation from the enzyme complex catalyzed by a thioesterase. The lactonization is possible either together with the liberation or immediately after. Both steps – aromatization and lactonization – are most likely spontaneous reactions without the necessity of any enzymatic support [44].

The validity of this putative biosynthetic pathway has been supported by a biomimetic total synthesis of alternariol, where 3,5,7,9,11,13-hexaoxotetradecanoic acid (corresponding to the penultimate product in **Fig. 9**) could be cyclized to the natural product, albeit with low yield [44]. A short and high-yielding total synthesis of alternariol with a biaryl coupling as the key step has been published quite recently [45].

Here, we identified the polyketide synthase, PksJ, involved in alternariol biosynthesis in *A. alternata*. PksJ is predicted to be a 2225 amino acid long, multifunctional iterative type I RD-PKS with KS, AT, DH, ER, KR and ACP domains. The expression of the *pksJ* gene appeared to be highest from day seven onwards when compared to other PKS genes, which correlated well with AOH production. PksJ carries a peroxisomal targeting signal type 1 (PTS1) at the C-terminal end, which suggests that AOH biosynthesis takes place in peroxisomes. This is not the first example for a role of peroxisomes in secondary metabolite biosynthesis. Recently, this has been shown for an enzyme of the AK toxin biosynthesis in *A. alternata* and also for penicillin biosynthesis [46], [47], [48].

To elucidate the function of PksJ, the gene was deleted and downregulated by siRNA. In both cases, AOH and AME were significantly reduced. RNAi knockdown transformants of phytonyl CoA dioxygenase (J-DI) and hydrolase (J-HL), flanking *pksJ* (1.34 kb upstream and 3.21 kb downstream of the *pksJ* gene, respectively), did not show any significant difference in the AOH and AME level as compared to wildtype. Thus these two genes probably are not involved in alternariol biosynthesis. However, the knockdown of the putative transcription factor (AltR) (11 kb upstream of *pksJ*) showed significantly lower levels of AOH and AME (**Fig. 8**). From this we conclude that AltR and PksJ are sufficient for AOH production. The gene encoding the methyl transferase located 15 kb upstream of *pksJ* could also be involved in AME formation. This enzyme consists of 313 amino acids with a calculated molecular mass of 38 kDa. This value is in good agreement with data for the purified enzyme. It displayed an apparent molecular mass of 43 kDa [15].

Another interesting outcome of this work is the finding that a second gene cluster, the *pksH* gene cluster, appears to influence the biosynthesis of AOH. *pksH* is predicted to be 8466 bp long with the capacity to encode a 2821 amino acids long protein. PksH has the same domain archaeology as PksJ with an additional MeT domain and shares almost 76% similarity with lovastatin nonaketide synthase of *Pyrenophora tritici-repentis* (Pt-1C-BFP). The reduction of the AOH level due to downregulaton of *pksH* could be explained if PksH would be required for some steps of the polyketide synthesis. Thus two PKS enzymes would contribute to one product. There are several examples that two gene clusters are involved in the synthesis of a given secondary metabolite, such as lovastatin (*Aspergillus terreus*), compactin (*Penicillum citrinum*), and T-toxin (*Cochliobolus heterostrophus* [31]. However, the fact that the reduction of the *pksH* level caused reduced expression of *pksJ* indicates rather an indirect effect through PksJ. Likewise, *pksH* downregulation also affected *pksI* expression. The fact that also downregulation of three other enzymes of the *pksH* gene cluster, a cytochrome P450 (H-cP450) enzyme, a transferase (H-TF) and a cyclin like F-box domain (H-CL) protein, caused downregulation of *pksJ* speaks also for a regulatory role of the *pksH* cluster. This hypothesis of regulatory role of this secondary metabolite appears quite attractive in terms of a putative application for mycotoxin production control. The understanding of the regulation of secondary metabolite formation through low molecular weight compounds could also be of importance for other economically important metabolites such as pharmacologically active compounds. It will be a challenge for future research to unravel such regulatory mechanisms.

### Conclusion

Alternariol is an important mycotoxin in food and feed contamination, but the elucidation of the biosynthesis and genetic control of the production has been essentially neglected so far. There are attempts to monitor alternariol concentrations in food within the European community and set standards for tolerated concentrations. The identification of PksJ as the responsible polyketide synthase opens the possibility to develop PCR-based detection methods for gene expression analyses before AOH is actually detectable. This technique has been developed already to monitor ochratoxin and other mycotoxins [49].

In summary, we have identified the polyketide synthase responsible for alternariol production. Our results suggest that only one enzyme is required for the biosynthesis of this compound. In addition, we propose a second metabolite, which regulates the expression of alternariol and at least one other metabolite.

## Methods

### Culture conditions and harvesting of spores of *Alternaria alternata*

*A. alternata* DSM 12633 cultures were grown in small petri dishes (60/15 mm) containing MCDB agar. For RNA analysis 5×10^4^ spores were inoculated in the liquid MCDB medium in small plates and incubated for one to twelve days at 28°C. For quantification of the spores they were harvested in sterile H_2_O, filtered for separation from mycelium with Miracloth (Calbiochem, Heidelberg) and centrifuged for 10 min. The pellet was resuspended in 1 ml of H_2_O and the number of spores was counted in a Helber chamber. The spore suspension was diluted with H_2_O and adjusted with 30% glycerol to give a suspension of 1×10^6^/ml and stored at −20°C. Strains are listed in **Table 1**.

**Table 1:** *Alternaria alternata* strains used in this study.

Strain	Genotype	Source
DSM 12633	Wildtype	DSMZ (Braunschweig)
SDS 2	DSM 12633 transformed with fusion product of *pksJ* LB::*hph*:: *pksJ* RB	This study
SDS 3	DSM 12633 transformed with fusion product of *pksH* LB::*hph*:: *pksH* RB	This study
SDS 6	DSM 12633 transformed with pDS 25; pSilent:: (p*)trpC*::*pksJ* sense::IT:: *pskJ* antisense:: (t)*trpC*:: *hph*	This study
SDS 7	DSM 12633 transformed with pDS 23; pSilent:(p*)trpC*::*pksH* sense::IT:: *pskH* antisense:: (t)*trpC*:: *hph*	This study
SDS 8	DSM 12633 transformed with pDS 88; pSilent::*(p)trpC*::*pksJ transcription factor (altR)* sense::IT::*pksJ transcription* antisense (*altR*)::*(t)trp*C, *hph*	This study
SDS 9	DSM 12633 transformed with pDS 72; pSilent::*(p)trpC*::*pksH cytochrome P450* sense::IT::*pksH cytochrome P450* antisense::*(t)trp*C, *hph*	This study
SDS10	DSM 12633 transformed with pDS 70; pSilent::*(p)trpC*::*pksH transferase* sense::IT::*pksH transferase* antisense::*(t)trp*C, *hph*	This study
SDS 11	DSM 12633 transformed with pDS 81; pSilent::*(p)trpC*::*pksH cyclin* sense::IT::*pksH Cyclin* antisense::*(t)trp*C, *hph*	This study
SDS 12	DSM 12633 transformes with pDS 74; pSilent::*(p)trpC*::*pksJ dioxygenase* sense::IT::*pksJ Dioxygenase* antisense::*(t)trp*C, *hph*	This study
SDS 13	DSM 12633 with pDS 78; pSilent::*(p)trpC*::*pksJ Hydrolase* sense::IT::*pksJ Hydrolase* antisense::*(t)trp*C, *hph*	This study

### Generation of fusion PCR fragments

Plasmids are listed in **Table 2**. Targeted gene deletion of *pksJ/pksH* was carried out according to the gene targeting procedure of Szewczyk and coworkers [28]. Briefly, in both cases, approximately one kb upstream and downstream of the corresponding ORF were amplified from *A. alternata* genomic DNA. Primers used in this study are listed in **Table 3** and **4** and were designed using the machine annotated draft genome sequence of the *A. alternata* strain ATCC 66981. In every case, the reverse primer for the 5′ region and the forward primer for the 3′ region carried 20 bp sequence tails that overlapped with the 5′and 3′ ends of the 3.9 kb *hygB* cassette. The *hygromycin B* resistance cassette was amplified from the pPK2 vector (kindly provided by N. Requena, Karlsruhe). The cassette consists of the *gpdA* promoter from *A. nidulans*, the hygromycin phosphotransferase gene from *E. coli* and the *trpC* terminator from *A. nidulans*. Three amplicons (the 5′ flanking region, the *hygB* cassette and the 3′ flanking region) were fused together by PCR using nested primers. As polymerase the phusion Polymerase (Thermo Scientific) was used. The purified product was directly used for transformation.

**Table 2:** Plasmids used in this study.

Plasmid	Construction	Source
pPk2	KanR, *(p)gpd*A, *hph*- Cassette, *(t)trp*C	[53]
pJet1.2/blunt Cloning Vector	Expressionsvector for directional cloning of PCR-products; AmpR, *rep* (pMB1), *eco47IR*	Fermentas
pSilent-1	gene silencing vector, AmpR, hph	[54]
pDS23	Silencing vector of *pksH*, pSilent::*(p)trpC*::*pksH* sense::IT::*pksH* antisense::*(t)trp*C, *hph*	This study
pDS25	Silencing vector of *pksJ*, pSilent::*(p)trpC*::*pksJ* sense::IT::*pksJ* antisense::*(t)trp*C, *hph*	This study
pDS70	Silencing vector for *pksH-transferase*, pSilent::*(p)trpC*::*pksH Transferase* sense::IT::*pksH Transferase* antisense::*(t)trp*C, *hph*	This study
pDS72	Silencing vector for *pksH-cytrochome P450*, pSilent::*(p)trpC*::*pksH Cytochrome P450* sense::IT::*pksJ –Cytochrome P450* antisense::*(t)trp*C, *hph*	This study
pDS74	Silencing vector for *pksJ-dioxygenase*, pSilent::*(p)trpC*::*pksJ* sense::IT::*pksJ –Dioxygenase* antisense::*(t)trp*C, *hph*	This study
pDS78	Silencing vector for *pksJ-hydrolase*, pSilent::*(p)trpC*::*pksJ* sense::IT::*pksJ –Hydrolase* antisense::*(t)trp*C, *hph*	This study
pDS81	Silencing vector for *pksH cyclin like F-Box domain*, pSilent::*(p)trpC*::*pksH Cyclin* sense::IT::*pksH –Cyclin* antisense::*(t)trp*C, *hph*	This study
pDS88	Silencing vector for *pksJ-transcription factor (altR)*, pSilent::*(p)trpC*::*pksJ Transcription factor* sense::IT::*pksJ Transcription factor* antisense::*(t)trp*C, *hph*	This study

**Table 3:** Primers used in this study.

Primer name	Sequence
**pksJ deletion**	
pksJ_LB_fwd_P1	AGCTCTGCAGGGTAGTCGAAGTCA
pksJ_P2	CTTCCAGCCAGACACGGTCC
pksJ_LB_rv_P3	*gaaattgttatccgctcaca*AAGGAATAAGGGTATGCAGTC
pksJ_RB_fwd_P4	*actggccgtcgttttacaac*GATTCGATAGTTAGCACTCTGG
pksJ_P5	GTCCGGGGCACTCTCAAG
pksJ_Rb_rv_P6	GATAGTTGTGGTTTGGGATATGC
pksJ_Lb_up_fwd	GTGGGAGCTGGGGGATG
hyg_gpd_rv	CGAAGACGTGGATCTTAACCAG
hyg_end_fwd	GTCCGAGGGCAAAGGAATAG
pksJ_Rb_down_rv	CGCTTACACATTACCATGGTTCTCTAC
**pksH deletion**	
pksH_LB_fwd_P1	GTGCCGTAGAGTAACCATTC
pksH_LB_rv_P3	*gaaattgttatccgctcaca*CATCAGCTAGGCGTAAAGAG
pksH_RB_fwd_P4	*actggccgtcgttttacaac*GGCTTATGCATGAATTGCTAATCTTC
pksH_RB_rv_P6	GACTTTTCGACTGCAGGTCTC
pksH_LB_up_fwd	GACTCAATGAAGCCGTCCTTG
pksH_RB_down_rv	GAGGCGACGAACATGTAGAG
**pksJ RNAi**	
pksJ_*Xho*I_fw_N	*ctcgag*GACTCGATACAGCCATTGCAG
pksJ_*HIn*dIII_rv	*aagctt*GTGCCTCCATACCCGAATGAG
pksJ_*Kpn*I_fw	*ggtacc*GACTCGATACAGCCATTGCAG
pksJ_*Bgl*II_rv	*agatctGTGCCTCCATACCCGAATGAG*
pksJ_RT_fw_N	GTCCCAAATTCCTACCCTCAC
pksJ_RT_rv__N	GATAGCCATCGAAAGCATTCCC
**pksJ hydrolase RNAi**	
pksJ_HL_*Xho*I_fw_N	*ctcgag*GAACGGCGAGTTGGACTTTG
pksJ_HL_*Hind*III_rv	*aagctt*AACACCCAAACCAGCCACAC
pksJ_HL_*Kpn*I_fw	*ggtacc*GAACGGCGAGTTGGACTTTG
pksJ_HL_*Bgl*II_rv_N	*agatct*AACACCCAAACCAGCCACAC
pksJ_Hl_RT_fw	ACGCCTTCGCCAATCATCTC
pksJ_HL_RT_rv	AACACCCAAACCAGCCACAC
**pksJ_dioxygenase RNAi**	
pksJ_DI_*Xho*I_fw	*ctcgag*CATAAGCGTGCTGCTATTGAGAAG
pksJ_DI_*HIn*dIII_rv	*aagctt*CTACCAGTCCTTCGCAAGTCTG
pksJ_DI_*Kpn*I_fw	*ggtacc*CATAAGCGTGCTGCTATTGAGAAG
pksJ_DI_*Bgl*II_rv	*agatct*CTACCAGTCCTTCGCAAGTCTG
pksJ_DI_RT_fw	GAGTATAAAGCACCAGACAAACG
pksJ_DI_RT_rv	TCCTCTCGTCATACCTCTCC

Orientation 5′ to 3′. Overhangs and restriction sites are in italics and in lower case.

**Table 4:** Primers used in this study.

Primer name	Sequence
**pksJ transcription factor RNAi (AltR)**	
pksJ_Tfact_*Xho*I_fw	*ctcgag*GCGCGACGGAAAGTATCTAGGAC
pksJ_Tfact_*Hin*dIII_rv	*aagctt*GCGGTGTTTGCTCGATACTACC
pksJ_Tfact_*Kpn*I_fw	*ggtacc*GCGCGACGGAAAGTATCTAGGAC
pksJ_Tfact_*Stu*I_rv	*aggctt*GCGGTGTTTGCTCGATACTACC
pksJ_TF_RT_fw	CAATCCAATCCAAACGCCATC
pksJ_TF_RT_rv	TGTCCAACCCATCCATCTCC
**pksH RNAi**	
pksH_*Xho*I_fw	*ctcgag*GATGCGGAAGCACTCCCTAC
pksH_*Hin*dIII_rv	*aagctt*CTAGTCCGCTGACCGGTTC
pksH_*Kpn*I_fw	*ggtacc*GATGCGGAAGCACTCCCTAC
pksH_bglII_rv	*agatct*CTAGTCCGCTGACCGGTTC
pksH_RT_fw_N	GTCAACCCTCTCACACCAAC
pksH_RT_rv_N	GACGCATCGCTTCAATAGCC
**pksH cytP450 RNAi**	
pksH_p450_*Xho*I_fw	*ctcgag*CTGACGAAACCTCTTCTCACAAG
pksH_p450_*Hin*dIII_rv	*aagctt*CAGCTCCAAACGGTGTCGAC
pksH_p450_*Kpn*I_fw	*ggtacc*CTGACGAAACCTCTTCTCACAAG
pksH_p450_*Bgl*II_rv	*agatct*CAGCTCCAAACGGTGTCGAC
pksH_p450_RT_fwd_N	GGGCTTTTGACTTTGAGCT
pksH_p450_RT_rv_N	CATCTCCCGTTCTTTGAACC
**pksH transferase RNAi**	
pksH_TF_ *Xho*I_fw	*ctcgag*GGATTACTCGCCCTCGTCTC
pksH_TF_*Hin*dIII_rv	*aagctc*GATCGATTGGTGGCCGTTGTG
pksH_TF_*Kpn*I_fw	*ggtac*GGATTACTCGCCCTCGTCTC
pksH_TF_*Bgl*II_rv	*agatct*GATCGATTGGTGGCCGTTGTG
pksH_TF_RT_fw	ATGCTACCGATCCAATCAAACTC
pksH_TF_RT_rv	CGCCACAGTCAAAACGAACAG
**pksH cyclin RNAi**	
pksH_CL_*Xho*I_fw	*ctcgag*GCCGCTATCCATCGACTCCGTG
pksH_CL_*Hin*dIII_rv	*aagctt*CTCCACAGCTGACCGTCGAAGC
pksH_CL_*Kpn*I_fw	*ggtacc*GCCGCTATCCATCGACTCCGTG
pksH_CL_*Bgl*II_rv	*agatct*CTCCACAGCTGACCGTCGAAGC
pksH_Cl_RT_fw	GAAGACACTCGCTGAAGCTC
pksH_CL_RT_rv	CGATGCGAAGACCAACCTC
benA fw	GTTGAGAACTCAGACGAGACCTTCTGCATTG
benA rv	GAACCATGTTGACGGCCAACTTCCTC

Orientation 5′ to 3′. Overhangs and restriction sites are in italics and in lower case.

### Protoplast transformation of *A. alternata*

The transformation procedures were based on the protocol of *A. brassicicola* [50] with modifications. Fungal spores were harvested from a MCDB culture plate, filtered, inoculated in 100 ml Richard's Liquid Medium (sucrose 20 g/l, KNO_3_ 10 g/l, KH_2_PO_4_ 5 g/l, MgSO_4_×6 H_2_O 2,5 g/l, yeast extract 1 g/l) and incubated for 19–24 hours at 30°C and 150 rpm. Mycelium was harvested by filtering, washed with 0.7 M NaCl and digested with Kitalase (Wako Chemicals) (60 mg in 6 ml 0.7 M NaCl) for 1 hour under soft shaking at 80 rpm and 30°C. Protoplast quality and quantity were checked microscopically. Protoplasts were separated by filtering through miracloth and glass wool and washed with 0.7 M NaCl by centrifugation at 7000 rpm, 4°C, 10 minutes, followed by a second washing step with STC (1 M Sorbitol, 50 mM CaCl_2_, 50 mM Tris-Cl pH 8.0). The pellet was resuspended in 200–500 µl STC and protoplasts were counted in a Helber chamber. 4×10^6^ protoplasts were incubated for 1 minute at 37°C before 4 µg of DNA were added and the suspension further incubated on ice for 30 minutes. Cells were heat-shocked for 2 minutes at 42°C and after addition of 2 ml PEG (40% PEG 4000, 50 mM Tris-HCl pH 8.0, 50 mM CaCl_2_) incubated for 20 minutes at room temperature. The suspension was spread on regeneration medium containing hygromycin (1 M sucrose, 0.5% caseic acids, 0.5% yeast extract, 80 µg/ml hygromycin) and incubated for 3 days at 28°C.

### Expression analysis

All samples were harvested from mycelium grown on MCDB liquid culture for 12 days (or the time indicated in the figures) in the dark at 28°C, pooled, frozen at −80°C, and lyophilized overnight. Total RNA was extracted from mycelia using Qiagen RNeasy Plant mini-kit (Qiagen, Hilden) following the manufacturer's instructions and purified by treatment with DNaseI (Invitrogen). RNA was diluted to 50 ng/µl and used as a template for quantitative RT-PCR, which was performed on a Bio-Rad iCycler MyIQ using the SensiFAST SYBR and Fluorescein Kit (Bioline, Germany). For each sample, three replications were performed. Each reaction mixture (20 µl) contained 2 µl of RNA template, 10 µl of 2× SensiFAST SYBR & Fluorescein Mix, 0.2 µl Reverse Transcriptase, 0.4 µl Ribosafe RNase Inhibitior, 5.4 µl of H_2_O and 2 µl of primer mix (each primer-5 mM). All samples were normalized using benA fw and benA rv primers as a control, and the values were expressed as the change relative to the levels of the control sample. Data analysis was performed with the IQ5 optical system software version 2.0.

### Analysis of mycotoxins using thin layer chromatography (TLC) and LC/MS

For the extraction of mycotoxins, three disks from each plate were excised with the back of a blue pipette tip and extracted by shaking with 1 ml ethyl acetate for 1 hour. The solvent was vaporized in a speed vac and the pellet resolved in 60 µl ethyl acetate. 20 µl were used for TLC with a mobile phase composed of toluol, ethylacetate and formic acid (5∶4∶1) on silica plates (Merck TLC silica gel 60) and visualized under UV light, 365 nm. As a standard, TLC-prepared AOH was used. For LC/MS analysis the same extracts were used but the ethyl acetate was evaporated and the pellet resolved in 100 µl methanol.

### LC-DAD-MS analysis

A LXQ Linear Ion Trap MSn system (Thermo Fisher Scientific, Waltham, Massachusetts, USA) together with a Finnigan Surveyor HPLC system equipped with a binary pump, autosampler, DAD and Xcalibur 2.0.7 software for data collection and analysis was used. This allowed on-line analysis of UV absorption and MS. 10 µl sample extract were injected. Separation was carried out on a 250×4.6 mm id, 5 µm, reversed-phase Luna C8 column (Phenomenex, Torrance, California, USA). Solvent A was deionized water and solvent B was ACN (both containing 0.1% formic acid). A gradient was started at 40% B, after 2 min changing from 40% B to 50% B in 5 min, then to 70% B in 5 min. After 12 min at 70% B, it went to 100% B within 5 min. After eluting the column with 100% B for 4 min, the initial 40% B were reached in 1 min, followed by conditioning of the column for 4 min. The flow rate was 0.5 ml/min. The mass spectrometer was operated in the negative ESI mode. Nitrogen was used as sheath gas, auxiliary gas and sweep gas with flow rates of 26.0, 15.0 and 0.02 l/min, respectively. Spray voltage was 4.0 kV, spray current 0.04 µA, capillary voltage −45.0 V, capillary temperature 350°C and tube lens voltage −125 V. For MSn analysis, CID voltage was set to 2.5 V.

### Generation of RNAi plasmid constructs

For construction of the *pksJ/pksH* RNA-silencing vector, we used PSilent-1 vector developed by Nakyashiki et al. [51] which can be used for a wide range of ascomycetes. The pSilent-1 vector carries the *A. nidulans trpC* gene promoter and terminator for expression of the hairpin cassette, and a hygromycin resistance gene for selection of the transformants. Briefly, approximately, 500 bp of the β-ketoacyl synthase (KS) region, specific to *pksJ/pksH* were amplified from cDNA by PCR using a primer set of either pksJ-*Xho*I_fw_N/pksH-*Xho*I_fw, containing a *Xho*I site, pksJ-*Hin*dIII_rv_N/pksH-*Hin*dIII_rv containing a *Hin*dIII site or pksJ-*Kpn*I_fw/pksH-*Kpn*I_fw containig a *Kpn*I siteand pksJ-*Bgl*II_rv/pksH-*Bgl*II_r containg a *Bgl*II site. PCR products were subcloned into pJET 1.2 (Fermentas Life Sciences). Both subcloned PCR products, amplified with pksJ-*Xho*I_fwd_N/pKsH-*Xho*I_fwd and, pksJ*Hin*dIII_rv_N/pksH-*Hin*dIII_rv and pSilent-1 vector were digested with *Xho*I and *Hin*dIII and ligated at the multiple cloning site of the pSilent-1 vector in the region between the *trpC* promoter and the spacer region. The vector was redigested with *Kpn*I and *Bgl*II, and the PCR product amplified with pksJ-*Kpn*I_fw and pksJ-*Bgl*II_rv was subcloned after digestion with *Kpn*I and *Bgl*II at the region between the spacer region and the *trpC* terminator. Insertions and direction of respective PCR products were confirmed by sequencing. The same method was followed for the other silencing constructs.

### Southern Blot and diagnostic PCR

Genomic DNA was isolated from mycelium of wildtype and the transformant grown on 28°C on MCDB liquid culture. Restriction enzyme digestion of genomic DNA (10 µg), agarose gel electrophoresis and transfer to Roti® Nylon plus membrane (Carl Roth, Germany) by capillary transfer were conducted under standard conditions [52]. PCR probes for Southern blots were prepared using the PCR DIG synthesis kit (Roche, Mannheim, Germany), with respective primer sets, following the manufacturer's instructions. Southern hybridization was performed at 68°C overnight with digoxigenin (DIG) labeled probes. Diagnostic PCR was performed using one external primer and a primer located inside the marker gene. The deletion strains yielded a PCR product of the expected size, whereas no product was present in the wildtype.
